# Optimal Control of Colloidal Trajectories in Inertial Microfluidics Using the Saffman Effect

**DOI:** 10.3390/mi11060592

**Published:** 2020-06-15

**Authors:** Felix Rühle, Christian Schaaf, Holger Stark

**Affiliations:** Institut für Theoretische Physik, Technische Universität Berlin, D-10623 Berlin, Germany; christian.schaaf@tu-berlin.de

**Keywords:** inertial microfluidics, optimal control, Saffman effect, 47.11.+j, 47.60.Dx

## Abstract

In inertial microfluidics colloidal particles in a Poiseuille flow experience the Segré-Silberberg lift force, which drives them to specific positions in the channel cross section. An external force applied along the microchannel induces a cross-streamline migration to a new equilibrium position because of the Saffman effect. We apply optimal control theory to design the time protocol of the axial control force in order to steer a single particle as precisely as possible from a channel inlet to an outlet at a chosen target position. We discuss the influence of particle radius and channel length and show that optimal steering is cheaper than using a constant control force. Using a single optimized control-force protocol, we demonstrate that even a pulse of particles spread along the channel axis can be steered to a target and that particles of different radii can be separarted most efficiently.

## 1. Introduction

The field of microfluidics is of utmost importance for numerous technological, biochemical, and biomedical applications, especially for inexpensive lab-on-a-chip applications and parallelized or automized studies [1,2,3,4]. The experimental realization of high throughput has led to the emergence of inertial microfluidic systems [5,6], that open up new possibilities. One important characteristic of the regime of intermediate Reynolds numbers, where flow is still laminar, is the breaking of Stokes reversibility. This leads to self-assembly, such as the famous Segré-Silberberg effect discovered by its namesakes in 1961 [7], where colloids travel to distinct lateral positions in the channel cross section that are driven by inertial lift forces. Exploiting secondary flow and inertial effects leads to many exciting applications [5], such as enhanced micromixing in curved channels [8], particle separation and filtration [9,10], or focusing and self-assembly [11,12,13]. An intriguing aspect is the reaction to external forces pointing along the axial direction of the microchannel. Under creeping flow conditions, these forces do not cause lateral migration [14], but do so in inertial microfluidic based on the so-called Saffman effect [15]. Thus, the particles’ lateral positions in the cross section of a microchannel can be manipulated with axial external forces, which drive the particles, e.g., via electrophoresis [16,17,18].

Parallel to the experimental progress, there have been continuous and fruitful efforts to tackle inertial microchannels via computer simulations [19]. Here, lift forces and particle dynamics can be probed for different channels and particle types [20,21,22], or in complex fluids [23,24]. Using external forces, optimal and feedback control has been applied to particle separation and steering under inertial microfluidic conditions [25,26], also together with thermal noise. One example is the hysteretic control scheme, as applied by Prohm and Stark [26]. Here, particles are periodically forced back to the channel center while using the Saffman effect, while the inertial lift force drives them away from the center. Thus, the particle stays within a finite interval around the channel center.

**Figure 1:**
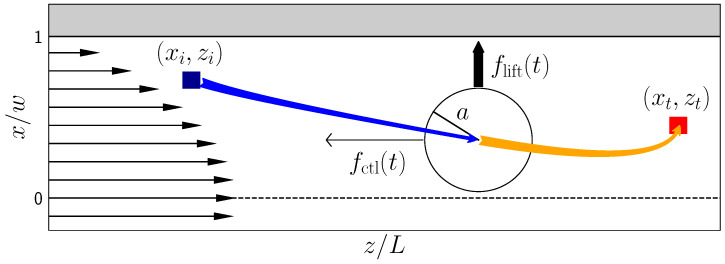
Sketch of the model system: a colloid flowing in the *x*-*z* plane experiences an axial control force $f_{\mathrm{ctl}}\left(t\right)$. The occuring Saffman effect changes the lateral lift force $f_{\mathrm{lift}}$ and, thereby, the colloid can be steered from the initial position $\left(z_{i},x_{i}\right)$ to the target $\left(z_{t},x_{t}\right)$.

In this article, we present the theoretical concept to realize precise particle steering with time-dependent axial control forces. The main idea is to steer the particles to different outlets of a microchannel in order to achieve particle separation and filtration. We use the Saffman effect and optimal control theory [27] to design the time protocol of the axial control force in order to steer particles from an initial to a target position in a microchannel (see Figure 1). These positions are defined, e.g., by inlets and outlets of the microchannel. As an input for the optimization, we employ the lattice-Boltzmann method to simulate particles in Poiseuille flow in order to obtain a whole set of lift-force profiles, depending on the axial control force. Subsequently, we use analytical fit functions to set up a system of ordinary differential equations that yield the particle trajectories. The time-dependent axial control force for optimally steering the particle from an inlet to an outlet follows by numerically minimizing a cost functional with respect to the control force under the condition that the target at the end of the channel is reached. We thoroughly discuss this method of optimal steering and compare it with steering by a constant control force. Using the optimal control-force protocol for a single particle, we demonstrate that even a pulse of particles spread along the channel axis can be steered to a target. Finally, we show how a single optimized control-force protocol can separate particles of different radii. Here, we go beyond particle separation with constant axial forces suggested in ref. [26]. One can further increase the lateral particle distance at the end of the channel by using the same time-dependent control force for both particles.

We introduce the theory of inertial microfluidics and the Saffman effect in Section 2. We describe the setup of our system, the lift force profiles, and the method of optimal control in Section 3. The results of our study for single and multi-particle steering are presented in Section 4 and we conclude in Section 5.

## 2. Theory—Inertial Microfluidics

Segré and Silberberg first reported how colloidal particles self-organize on an annulus under pipe flow conditions [7] that is located approximately halfway between the channel center and the confining walls. Because deterministic lateral motion for rigid particles is impossible under strict creeping flow conditions, this migration results from the inertial term of the Navier–Stokes equation. Hence, it was termed inertial focusing and rationalized by a so-called inertial lift force [5,28,29]. For channels with rectangular cross sections, these equilibrium positions are either located on the main axes or the diagonals of the cross section, which depends on the particle radius and the cross-sectional aspect ratio (see, for example, ref. [26]). If this ratio is sufficiently large, only two stable positions on the short axes exist and it is sufficient to treat the flowing particle in a two-dimensional plane, as sketched in Figure 1.

The Poiseuille-flow profile in a rectangular channel is known analytically [30]. Because the flow field along the channel axis obeys $u=u\left(x,y\right)e_{z}$, the convective term of the Navier–Stokes equations vanishes, and for the stationary case the Stokes equations are recovered. Restricting fluid flow in the cross section to x∈(−w,w), y∈(−h,h) with h>w and using no-slip boundary conditions at the channel walls, u(x=±w,y)=u(x,y=±h)=0, one can write the solution as a Fourier series expansion [30]

(1) $$u\left(x,y\right)=\frac{16w^{2}\Delta p}{\pi^{3}\eta L}\sum_{n=0}^{\infty }{\left(-1\right)}^{n}\frac{1}{{\left(2n+1\right)}^{3}}\left[1-\frac{\cosh\left(\frac{(2n+1)\pi }{2w}y\right)}{\cosh\left(\frac{(2n+1)\pi }{2w}h\right)}\right]\cos\left(\frac{(2n+1)\pi }{2w}x\right).$$

Here, a constant pressure gradient Δp/L is used and the dynamic viscosity of the fluid η. When we employ this analytical formula in our numeric calculations, we truncate the series after n=100. The maximum flow velocitiy $U_{m}$ is reached at the center (x,y)=(0,0) of the channel. It is determined by the choice of the Reynolds number Re=ρUm2w/η, where ρ is the fluid density and 2w is the width of the channel.

Inertial effects become observable if a colloid is subjected to a Poiseuille flow at finite Reynolds numbers Re. This initiates a lift force acting on the colloid, which can be controlled via the Saffmann effect by applying an additional axial control force (see Figure 1). We introduce those in the following.

### 2.1. Lift Force

**Figure 2:**
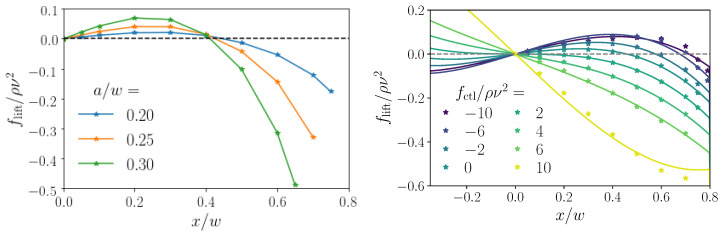
(**Left**): lift-force profiles along the positive *x* axis for different particle radii a/w at zero axial control force, $f_{\mathrm{ctl}}=0$. Note the larger strength of the lift forces for larger colloids and the shift of the stable fixed point. The force unit $\rho \nu^{2}$ uses fluid density ρ and kinematic viscosity ν=η/ρ. (**Right**): lift-force profiles for a colloid with radius a=0.2w at different axial control forces and least-square fits using Equation (4) (solid lines). In both cases, the Reynolds number Re=10 is used.

Since the discovery of inertial focussing different scaling laws for the dependence of the inertial lift force on particle radius *a* and Reynolds number have been derived [11,28,29,31,32]. For example, Ho and Leal calculated the lift force for small particle radius (a≪w) and small particle Reynolds number Re(a/w)2. They arrived at the scaling law flift∼Re2(a/w)4 [31], whereas numerical simulations at finite particle sizes arrived at $f_{\mathrm{lift}}∼{\left(a/w\right)}^{3}$ in the channel center for particle sizes a<w [5]. Often, the lift coefficient f(a,Re) is introduced to correct for finite particle size and Reynolds number [5]. In particular, it has been observed that the scaling exponent for the lift force as a function of the particle radius depends on the lateral position in the channel [5,11]. Importantly, the fixed points of the lift-force profiles indicating stable equilibrium positions change considerably with the geometry of the channel cross section (see, for example, ref. [26]). In the following, we numerically calculate the lift-force profiles using lattice-Boltmann simulations, as shortly explained in Section 3.2. Typical examples for a zero axial control force are presented in Figure 2, left.

The net inertial lift force is often described as a balance of two contributions. They arise from a stresslet that is the leading force distribution on the particle surface under a shear-flow gradient [6,31]: the reflection of the stresslet from the channel wall induces a force, which pushes the particle away from the wall, whereas the interaction with the shear gradient transports particles to regions of larger shear, which is towards the wall in the case of a Poiseuille flow.

### 2.2. Saffman Effect

**Figure 3:**
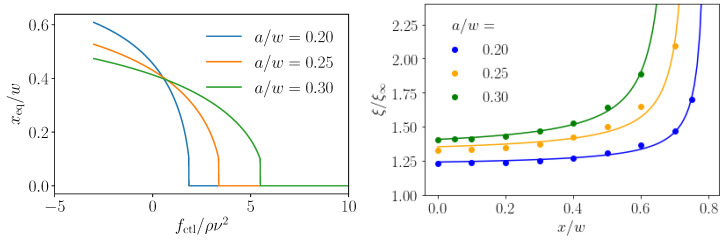
(**Left**): stable equilibrium positions (fixed points) as a function of control force for different particle radii a/w. They were determined using the analytical fits to the relevant lift-force profiles in Figure 2, right. (**Right**): hydrodynamic friction coefficients relative to the bulk value $\xi_{\infty }=6\pi \eta a$ plotted versus the lateral particle position for different particle radii. The solid lines are fits using Equation (4).

Applying an additional axial control force to the colloidal particle speeds up or slows it down relative to the local Poiseuille flow velocity. This modifies the slip velocity field close to the particle surface and, at finite Reynolds numbers, creates an additional lateral contribution to the lift force, described by Saffman [15]. It depends on the shear rate γ, rather than the shear gradient, and it was calculated to be $f_{S}∼va^{2}\gamma^{1/2}$ in bulk at small Reynolds numbers, where *v* is the difference between local flow field and particle velocity. Figure 2, right, demonstrates how the lift-force profile changes when a control force is applied. The stable fixed point ($f_{\mathrm{lift}}=0$ with a negative slope) moves from the zero-force position ($f_{\mathrm{ctl}}=0$) either to the wall or to the channel center, depending on whether the control force is applied along the flow direction ($f_{\mathrm{ctl}}<0$) or against it ($f_{\mathrm{ctl}}>0$), respectively. The respective stable equilibrium positions are plotted in Figure 3, left, also for different particle radii. Because the inertial lift force grows more strongly with the radius than the Saffman force, higher axial control forces are necessary to move the fixed point for larger particles towards the center. Consequently, the curves in Figure 3, left, become flatter for larger particle radii.

## 3. Methods

### 3.1. Setup

We consider a rectangular microchannel with Poiseuille flow at Reynolds number Re=10. The channel has an aspect ratio w:h of 1:2, where x∈(−w,w) and y∈(−h,h). The length of the channel is *L* with z∈(0,L), which we vary in the following. At such an intermediate Reynolds number, the regime of inertial microfluidics is reached and solid colloids in a Poiseuille flow self-organize towards distinct lateral focus positions [15]. For the aspect ratio chosen here, they equilibrate to the plane y=0 and, therefore, we only consider the particle dynamics in the *x*-*z*-plane [6,9,33]. Indeed in our lattice-Boltzmann simulations, the position y=0 is stable, i.e., a particle is immediately driven back once it leaves the center plane. The stable fixed point at this location was numerically determined in ref. [26]. Switching on the axial force, the induced Saffman force is strongest along the *x* axis, since, in this direction, the velocity gradients are largest. Therefore, we expect the particle to stay in the center plane. The stable equilibrium positions on the *x* axis ($f_{\mathrm{lift}}=0$) depend on particle size as we show in Figure 2, left. There is also a dependence on Re2 [13], which we do not further explore here. These positions are reached after the colloid has been advected for a sufficiently large axial distance $L_{f}$ without any external forcing. Di Carlo and co-workers mention an estimate for this length [5,6],

(2) $$L_{f}=\frac{\pi \nu w^{2}}{f_{L}U_{m}a^{2}},$$

with the maximum flow velocity Um=νRe/(2w), kinematic fluid viscosity ν, and particle radius *a*. For our aspect ratio w/h=0.5, ref. [5] gives a lift coefficient $f_{L}=0.05$. Furthermore, using Re=10, and a/w=0.2, in Equation (2), we obtain the focus length $L_{f}\approx 314w$. Now, applying an additional lateral force along the *x* direction, one can optimally steer particles to any position on the *x* axis, as we showed in ref. [25].

Here, we propose an alternative strategy for optimal steering while using the Saffman effect. We apply an axial control force and thereby modify the lift-force profile as demonstrated in Figure 2, right. In Figure 3, we show how the stable equilibrium position now depends on the control force. Subsequently, the idea is to use a time varying axial control force, which can be realized, for example, by electromagnetic fields [17], for optimal steering. The goal is to optimally steer a particle from an inlet, which is located at the start position $\left(z_{i},x_{i}\right)$, towards a target $\left(z_{t},x_{t}\right)$, fulfilling a criterion of optimality, as we will outline below. To implement this approach, we first need lift-force profiles $f_{\mathrm{lift}}\left(x,f_{\mathrm{ctl}}\right)$ for different control forces as well as friction coefficients ξ(x) for different particle sizes. We determined them with the help of lattice-Boltzmann simulations, and approximated them with appropriate fit functions (Section 3.2). The lift force profiles are then used in dynamical equations for the particle motion, which we solve with explicit Euler integration to determine the optimal steering path (Section 3.3).

### 3.2. Profiles for Lift Forces and Friction Coefficients

Our lattice-Boltzmann simulations (including the immersed-boundary method) [34,35] of single colloids in a microchannel in the inertial regime are described in detail in refs. [13,26], where we also explain how to determine inertial lift forces for each particle position.

The simulated lift-force profiles for different particle radii and channel Reynolds number Re=10 are shown in Figure 2, left. They display the well-known behavior of inertial focusing: colloids are driven away from the unstable fixed point at the origin and towards their stable equilibrium positions (stable fixed points) between the channel center and the wall, which depend on the particle radius. As in ref. [25], we perform a least-square fit of the lift-force profiles to a third-order polynomial of odd degree together with a wall-repulsion term as the particle approaches the walls. Additionally, we now also apply this fit to the dependence of the lift force on the axial control force $f_{\mathrm{ctl}}$ using coeffients that are second-order polynomials in $f_{\mathrm{ctl}}$. Thus, the functional form for the fit of our lift-force profiles is as follows

(3) $$\begin{array}{ccc} f_{\mathrm{lift}}\left(x,f_{\mathrm{ctl}}\right) & = & \phi_{1}\left(f_{\mathrm{ctl}}\right)x+\phi_{3}\left(f_{\mathrm{ctl}}\right)x^{3}+\phi_{w}f_{w}\left(x\right)\\ \phi_{1}\left(f_{\mathrm{ctl}}\right) & = & a_{1}f_{\mathrm{ctl}}^{2}+b_{1}f_{\mathrm{ctl}}+c_{1}\\ \phi_{3}\left(f_{\mathrm{ctl}}\right) & = & a_{3}f_{\mathrm{ctl}}^{2}+b_{3}f_{\mathrm{ctl}}+c_{3}\\ f_{w}\left(x\right) & = & \frac{1}{x-\left(1+\delta \right)x_{w}}+\frac{1}{x+\left(1+\delta \right)x_{w}}, \end{array}$$

where $x_{w}=w-a$ and we use $\delta =10^{-3}$ for numerical stability. The fit function works well, in particular, for non-zero control force, as long as the region immediately at the wall is avoided, as Figure 2 demonstrates. Note that we did not attempt to include the dependence on particle radius in our fit function, but, instead, perform a separate fit for each particle size. We do this in order to limit the number of parameters.

We also determined the friction coefficients of the particles in the lattice-Boltzmann simulations and plot their values as a function of the lateral position in Figure 3, right, for three different colloidal radii. The presence of the walls is clearly visible. We fit the position-dependent friction coefficient ξ by the function

(4) $$\xi \left(x\right)=\xi_{\infty }\left(d_{1}+d_{2}\frac{a}{\left(w-a)-|x\right|}\right),$$

where $\xi_{\infty }=6\pi \eta a$ is the bulk friction coeffcient and $d_{1}$, $d_{2}$ are the fit parameters. The fits as solid lines are presented in Figure 3, right.

### 3.3. Dynamical System and Optimal Control

Using the axial control force $f_{\mathrm{ctl}}$, the fitted lateral lift force $f_{\mathrm{lift}}$, and the friction coefficient ξ, the overdamped motion of the steered particle in the Poiseuille flow profile u(x)=u(x,y=0) is governed by the following differential equations disregarding any thermal noise:

(5) $$\begin{array}{ccc} \dot{z} & = & u\left(x\right)+\frac{1}{\xi (x)}f_{\mathrm{ctl}}\left(t\right) \end{array}$$

(6) $$\begin{array}{ccc} \dot{x} & = & \frac{1}{\xi (x)}f_{\mathrm{lift}}\left(x,f_{\mathrm{ctl}}\left(t\right)\right). \end{array}$$

Here, *z* is the coordinate along the channel and *x* in lateral direction. The size of the suspended particle influences the lateral motion implicitly via the friction coefficient and the fitted function for the lift force, as described above. In axial direction we do not consider that a force-free colloid is slower than the streaming fluid but simply set this velocity to the Poiseuille flow velocity u(x). We note that the dynamics of the colloid is always confined to one half of the channel. At x=0, the lift force—and, hence, the total lateral force—is exactly zero; therefore, it is impossible for the colloid to cross the center line.

Solving these equations for a given time protocol $f_{\mathrm{ctl}}\left(t\right)$ of the axial control force, determines x(t) and z(t). We are looking for an optimal protocol $f_{\mathrm{ctl}}^{*}\left(t\right)$, which steers a particle as close as possible to the target $\left(z_{t},x_{t}\right)$ at end time $T^{*}$. Thus, we define the cost functional [27]

(7) $$J\left[f_{\mathrm{ctl}}\left(t\right),T\right]=\frac{c_{x}}{2}|x_{t}-x\left(T\right){|}^{2}+\frac{c_{z}}{2}|z_{t}-z\left(T\right){|}^{2}+\frac{\varepsilon }{2}\int_{t_{0}}^{T}{\left|f_{\mathrm{ctl}}\left(t\right)\right|}^{2}dt,$$

and obtain the optimal steering control force by minimizing the cost functional with respect to $f_{\mathrm{ctl}}\left(t\right)$:

(8) $$f_{\mathrm{ctl}}^{*}\left(t\right)=\underset{f_{\mathrm{ctl}}}{\arg\;\min}J\;.$$

For the total duration *T* of the trajectory, which is undetermined on the right-hand side of Equation (7), the algorithm also finds an optimum $T^{*}$. In concreto, we set $T^{*}=N\Delta t^{*}$, where $\Delta t^{*}$ is the time step of our time discretization, choose a constant *N*, and determine $\Delta t^{*}$ together with the force protocol by minimizing the cost functional. We always choose a control force that is constant in time as our initial function. When optimizing the single-particle trajectories, we choose N=500; while, when looking at a pulse of colloids in Section 4.3.1, we take N=2500, since a higher resolution in axial direction is required. The functional in Equation (7) includes a regularization term, where we integrate over the square of the total force, because otherwise arbitrarily large forces would be permissible. Keeping this regularization term low decreases the energy cost for steering the particle along a specific trajectory, for example, electrophoretically by applying an electric field [17]. We weigh the cost of deviating from the target area differently for the *x* and *z* coordinates, because the velocities differ strongly. The control parameters $c_{x},c_{z}$ and ε have to be manually adapted to find the right balance between the cost of higher forces versus the precision of steering. When optimizing the single-particle trajectories, we choose $c_{x}$ = 14,000, $c_{z}=0.3$, and ε=0.003. For solving the differential equations, we use explicit Euler integration. With this, we optimize the discretized cost functional $J[f_{\mathrm{ctl}}\left(t\right),T]$ while using a sequential quadratic programming (sqp) algorithm [36] provided by the package *fmincon* from MathWorks’ software matlab (Release R2019b, https://mathworks.com/help/optim/ug/constrained-nonlinear-optimization-algorithms.html). This robust and efficient method uses a Lagrangian representation of a constrained problem: $L=f\left(x\right)+\lambda^{T}c$, where *x* is the vector of unknowns, *c* the vector of equality constraints, and λ the vector of Lagrange multipliers. For the optimal solution $x^{*}$, the gradient of L vanishes, which yields a non-linear equation. Here, $x^{*}$ can be approximated by iteration, $x_{i+1}=x_{i}+d_{i}$, where the differences $d_{i}$ are determined in each step by a simpler approximated quadratic problem, for which standard quadratic solvers are available [36]. We do not calculate the necessary gradients to the functional ourselves, but leave this to the software matlab.

We also seek to maximize the lateral distance between two colloids, when approaching the target with axial coordinate $z_{t}$. Therefore, in the cost functional of Equation (7), we use $\frac{c_{x}}{2}|\Delta x\left(T\right)-\Delta x_{f}{|}^{2}$ for the first summand, where Δx(t) is the lateral distance between two colloids at time *t* and $\Delta x_{f}$ is the distance aimed for. In the second term involving the *z* coordinate, we add up the contributions from the two particles. The coefficient $c_{z}$ takes the same values for both colloids. For the two-particle optimization, we use $c_{x}=500$, $c_{z}=30$, and ε=0.01.

We always obtained smooth solutions when solving the unconstrained problem. It was not necessary to use a numerical constraint for the lateral coordinate *x* in order to keep the particle within the channel due to the diverging repulsive lift force close to the walls. The sqp algorithm required 75 iterations on average in order to converge for the single-particle steering. For the separation of two particles, 161 iterations were required on average.

## 4. Results and Discussion

In the following, we investigate steering strategies for single and multiple particles that make use of the aforementioned inertial lift forces. First, we discuss steering with a constant axial control force and then the outcome of our optimal control scheme. We investigate the implications for a particle pulse spread along the channel axis using the results from single-particle steering. Finally, we use optimal control to find control forces that maximize the separation of two particles so that they can be carried off at different outlets of a channel.

### 4.1. Steering with Constant Axial Control Forces

Exploiting the Saffman effect, the easiest approach to steer a particle to a target position is to use constant axial control forces. They shift the stable fixed point of the lift-force profile and, thereby, in principle, the equilibrium position of a colloidal particle can be adjusted arbitrarily, as shown in Figure 3. In the same way, also colloids of different sizes can be well separated within the microfluidic channel while using a constant control force [25]. As Figure 3 shows, this is achieved by choosing the control force, such that the smaller particle (e.g., a=0.2w) is pushed to the center while the larger particle still keeps a noticeable distance from the center.

**Figure 4:**
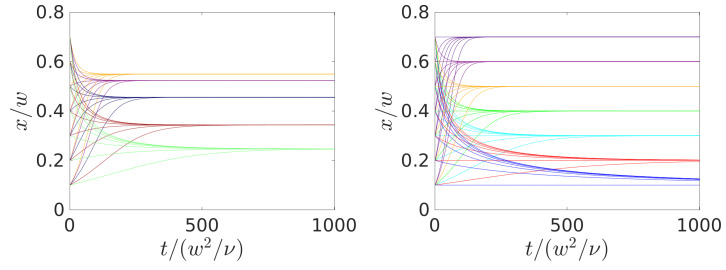
Inertial focusing of a colloid with radius a=0.2w starting from different initial positions and using constant control forces. Lateral position versus time is plotted. (**Left**): for constant control forces $f_{\mathrm{ctl}}/\left(\rho \nu^{2}\right)=-1.5,-1.0,0,1.0,1.5$ (which give an increasing equilibrium *x* position or stable fixed point). (**Right**): for adjusted constant control forces such that the stable fixed point assumes the values x/w=0.1,0.2,…,0.7.

In Figure 4, the lateral positions for the moving colloid are plotted versus time for specific control forces (left) and when the forces are adjusted to give specific final positions (right). Negative forces point along the direction of the channel flow and, thus, drive the equilibrium position closer to the wall, whereas positive forces slow down the colloid and thereby induce motion towards the channel center. A closer inspection of the two plots shows that the necessary travel time for focusing varies with the initial position. However, more pronounced is the dependence on the final position as Figure 4, right, demonstrates. Fixed points that are closer to the channel center are reached later than those closer to the wall. This is consistent with the fact that the Saffman effect (or the shear-induced lift force) increases with the shear rate, which is larger close to the walls. Thus, using the Saffman effect for steering requires less time the closer the final position is situated to the wall. In Section 3.1, we evaluated the focus length $L_{f}\approx 314w$ for our setup. Indeed, it gives a good estimate for the focus length in our simulations at zero control force. When plotting the lateral positions of Figure 4 versus the traveled axial distance *z* instead of time, the curves look very similar to the ones in Figure 4, even though particles closer to the center (small *x*) should flow faster and the curves should stretch even farther close to the centerline. However, to reach these targets, the control force has to act against the flow and, therefore, decreases the axial flow velocity of the particles.

In conclusion, in order to relax to the adjusted equilibrium position with the constant-force strategy, considerably longer travel times and axial distances are required for targets that lie close to the channel center. Therefore, long enough channels need to be used in order for the strategy to work. Thus, it is not possible to use a single channel length to steer particles to different lateral target positions using the constant-force strategy. Furthermore, steering with a constant axial force means that it has to be maintained for the whole trajectory. We compare the cost of this constant-force scheme with the optimal control scheme in the next section, which also allows for operating with channels of one length.

### 4.2. Optimal Control of Single Colloids

We now turn to the optimization problem for the cost functional $J[f_{\mathrm{ctl}}\left(t\right),T]$ of Equation (7) set up in Section 3.3. This will provide us with a time-dependent control force and the particle trajectory in the *x*-*z* plane. In the following, we provide optimal solutions of the cost functional for different start and end positions in the channel. The optimization procedure is applied to two axial target positions at $z_{t}=300w$ and $z_{t}=500w$. Furthermore, three particle sizes with radii a=0.2w, 0.25w, and 0.3w are considered. Because the initial position is always set at $z_{i}=0$, we call $z_{t}$ channel length for short.

**Figure 5:**
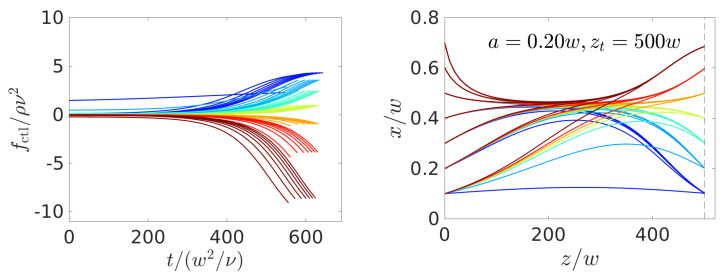
Optimal control-force protocols (**left**) and particle trajectories in the *x*-*z* plane (**right**) found for steering colloids with radius a=0.2w from a set of initial positions to a set of targets, which both assume the same values {0.1,0.2,0.3,0.4,0.5,0.6,0.7}. The same color refers to trajectories ending at the same target position $x_{t}$. The vertical dashed line indicates $z_{t}$.

In Figure 5, we plot the optimal force protocol (left) and the trajectories (right) resulting from different start and target positions while using the particle radius a=0.2w and channel length $z_{t}-z_{i}=500w$. Interestingly, the regularization term generates solutions, where the force is zero at first, meaning that all colloids travel towards the equilibrium position at zero control force except when both $x_{i}$ and $x_{t}$ are close to the channel center. In particular, for $x_{i}=x_{t}=0.1w$, the algorithm chooses a nearly constant force protocol, clearly recognizable in Figure 5, left. In all other cases, the control force increases or decreases monotonously starting around $t=300w^{2}\nu^{-1}$, which corresponds to a traveled distance between z=250w and 300w. At the end of the trajectory the target is reached with high precision at $z=z_{t}$.

Note that the control-force protocols are similar for the same target position (same color). However, the increase/decrease from zero starts earlier if the initial lateral position is closer to the channel center. This is because the flow velocity is larger and thus particles travel faster downstream.

**Figure 6:**
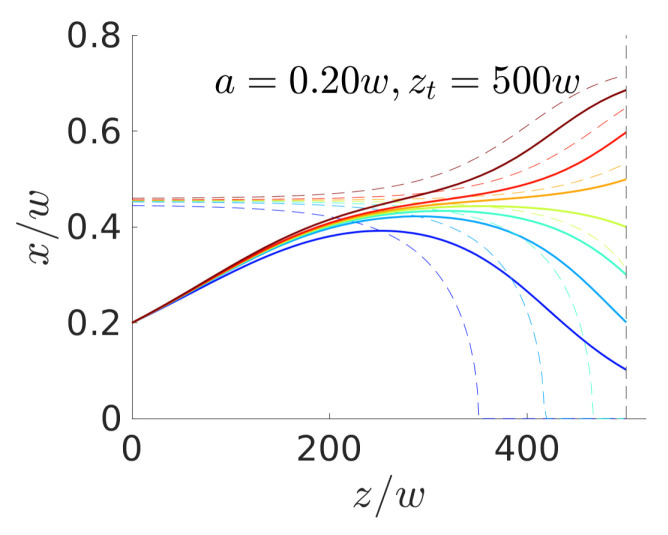
Solid lines: Optimal particle trajectories in the *x*-*z* plane found for steering colloids with radius a=0.2w from the initial position $x_{i}=0.2w$ to a set of targets {0.1,0.2,0.3,0.4,0.5,0.6,0.7}. Dashed lines: Sequence of fixed-point positions of the lift-force profiles resulting from the optimal control-force protocols $f_{\mathrm{ctl}}\left(t\right)$ (same colors represent the same force protocol).

From the explanation so far, one could assume that the particle instantaneously follows the stable fixed points of the lift-force profiles that are associated with the optimal control-force protocol $f_{\mathrm{ctl}}\left(t\right)$. We plot the sequence of fixed points as dashed lines in Figure 6 in the x,z plane together with the realized particle trajectories starting at $x_{i}=0.2w$ and ending at different target positions. Clearly, the particle does not follow the sequence of stable fixed points, since migrating there is hindered by viscous friction. For target positions that are close to the channel center, the control force induces a fixed point at the center (x=0) for the last part of the trajectory to accomplish the trajectories bend downward in Figure 6.

#### 4.2.1. Comparison with Constant-Force Strategy

**Figure 7:**
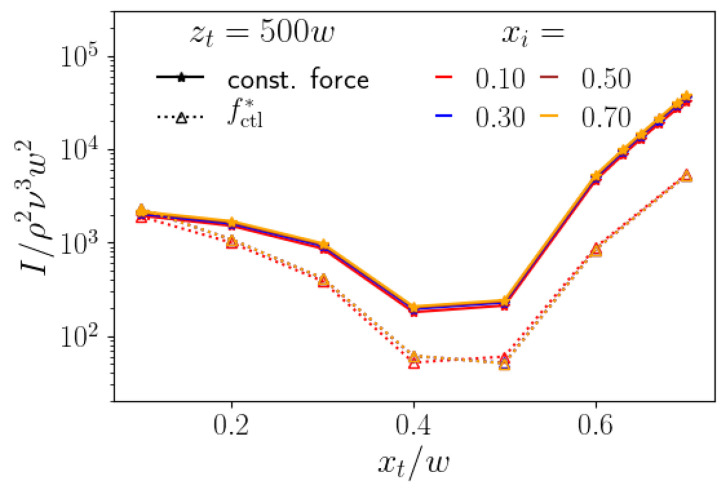
Comparison between the constant-force strategy (solid lines) and the optimal control-force strategy (dashed line), for different inital lateral positions and a channel length $z_{t}=500w$. We show a semi-logarithmic plot of the cost functional *I* of the axial control force versus lateral target position $x_{t}$.

We compare the costs of the constant-force strategy and the optimal control-force scheme while using the cost functional $I:=\int_{0}^{T^{*}}{\left|f_{\mathrm{ctl}}^{*}\right|}^{2}\left(t\right)dt$. It integrates the square of the control force along the particle trajectory, where lateral initial and target positions are equal for both strategies. Note that *I* is a measure for the energy costs needed to realize the control schemes. To compare both of the strategies, we decided to work with a constant channel length $z_{t}$ as a typical situation in experiments. While the optimal-control scheme can be adjusted to such a specific axial target $z_{t}$, for the constant-force strategy the necessary channel length varies, depending on the lateral target position, as we discussed in Section 4.1. In Figure 7, left, we plot the cost functional *I* for both strategies versus target position $x_{t}$ for different initial lateral positions $x_{i}$. The channel length is always $z_{t}=500w$. Interestingly, the curves for each strategy are all very similar and, therefore, independent of $x_{i}$. The reason is that the control-force profiles for different $x_{i}$ but same $x_{t}$ in Figure 5 all have very similar shape and are mostly shifted relative to each other. Clearly, the optimal-control scheme is less costly than the constant-force strategy up to an order of magnitude, besides for the smallest target position $x_{t}/w=0.1$. Here, we note that, for the constant-force strategy, the channel length $z_{t}=500w$ is not sufficient for reaching targets with $x_{t}/w\leq 0.4$. Taking the need for longer channels into account, the costs of the constant-force strategy goes up. In contrast, for large $x_{t}\geq 0.5$, a channel length that is smaller than 500w is sufficient, which reduces the costs. However, those do not fall below the costs of the optimal-control scheme.

#### 4.2.2. Dependence on Particle Size

**Figure 8:**
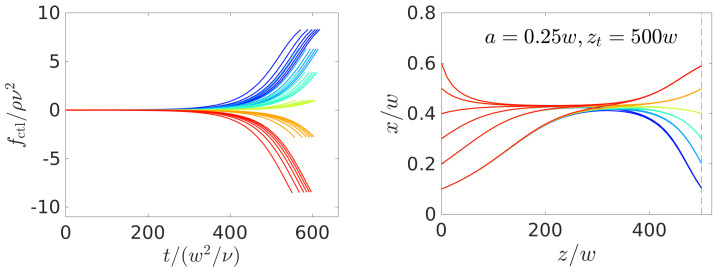

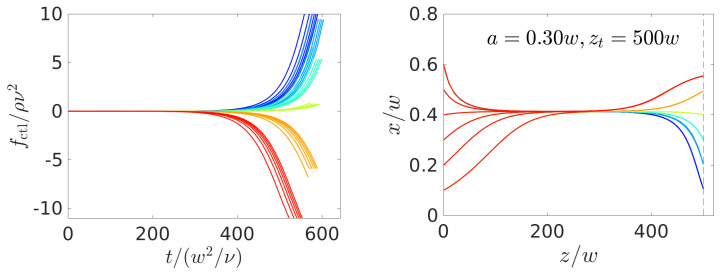
Optimal control-force protocols (**left** column) and particle trajectories in the *x*-*z* plane (**right** column) found for steering colloids with radius a=0.25w (**top** row) and a=0.3w (**bottom** row) from a set of initial positions to a set of targets, which both assume the same values {0.1,0.2,0.3,0.4,0.5,0.6}. The same color refers to trajectories ending at the same target position $x_{t}$. The vertical dashed line indicates $z_{t}$.

Increasing the particle size strongly increases the strength of the inertial lift force ($f_{\mathrm{lift}}\propto {\left(a/w\right)}^{4}$ for very small particles). Thus, the fixed-point position at the initially zero control force is reached faster, as a comparison of the force protocols and the particle trajectories in Figure 5 and Figure 8 for different radii shows. The control force remains longer at a zero value before it steers the particle to its target position. However, for the larger particles, this then also requires larger control forces to steer them laterally, because they experience a higher drag force and it is therefore harder to move them relative to the external flow. Furthermore, as before for target positions further away from the zero-force fixed-point position, larger control forces are necessary for steering, which makes sense. Finally, as we already noted in Section 2.2, since the Saffman force ($f_{S}\propto a^{2}$) grows less strongly with the radius than the focusing inertial lift force, one again needs larger control forces for particle steering, which also drives up the whole costs. Thus, in all trajectories, the particles utilize inertial focusing at first to relax towards the zero-force equilibrium positions and the control force is then switched on.

#### 4.2.3. Dependence on Channel Length

**Figure 9:**
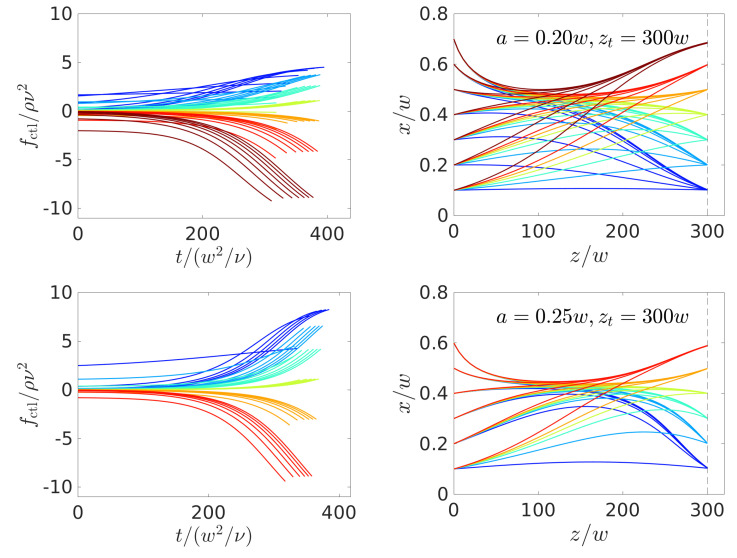

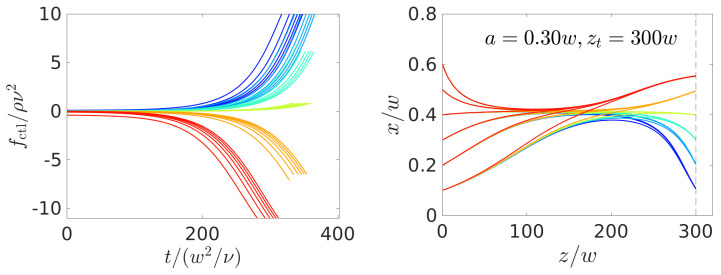
Optimal control-force protocols (**left** column) and particle trajectories in the *x*-*z* plane (**right** column) found for steering colloids with radii a/w=0.2, 0.25, and 0.3 (**top**, **middle**, and **bottom** row) to an axial target position $z_{t}=300w$. The vertical dashed line indicates $z_{t}$.

Because the control forces obtained in Figure 5 and Figure 8 all remain at zero at the beginning of the particle trajectories, it should be possible to further decrease the channel length. Indeed, we managed to obtain numerically stable solutions for a channel length of $z_{t}=300w$, which we show in Figure 9. Here, the trajectories do not (a/w=0.2 and 0.25) or only shortly (a/w=0.3) stay on the lateral focus position and, thus, the control force is always non-zero or zero for a short time. Interestingly, the algorithm chooses relaxation towards the equilibrium position for the largest radius a=0.3w. Again, the inertial lift force increases strongly with the particle radius and, therefore, it is too costly to compete against it with a non-zero control force over the whole simulation time. Instead, the algorithm chooses to drive up the control force to high absolute values, but for a shorter time period at the end of the trajectory.

### 4.3. Controlled Steering of Multiple Colloids

We use our model to steer two or more particles to their respective targets. As a first approximation, here we neglect two-particle interactions. It is known that the lift-force profiles of two particles are influenced due to secondary flows, when their axial distance is smaller or of the order of the channel width [6,13]. In the following, we first consider the steering of a pulse of equal-sized particles, and then investigate the lateral separation of two particles with different radii under the same control-force protocol.

#### 4.3.1. Steering a Pulse of Colloids

**Figure 10:**
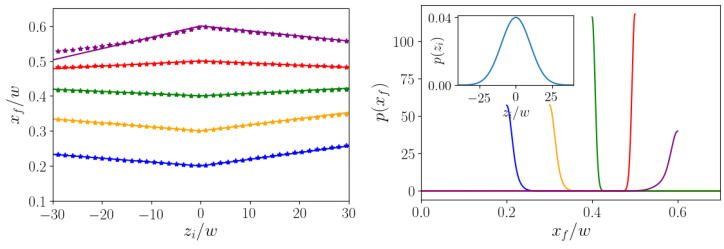
(**Left**): Final lateral position $x_{f}$ for particles with initial axial position $z_{i}$ when steered by the axial control force $f_{\mathrm{ctl}}^{*}\left(t\right)$, which is opimized for steering the central particle with $z_{i}=0$ and $x_{i}=0.2$ to different target positions $x_{t}/w=0.2$, 0.3, 0.4, 0.5, and 0.6 at the axial target position $z_{t}=500w$. The linear fits by solid lines are hardly visible. (**Right**): Distributions of final lateral positions $x_{f}$ for the Gaussian distribution of initial axial positions $z_{i}$ shown in the inset. The colors indicate different target positions $x_{t}/w$ as in the plot on the left.

In the following, we consider the situation where a pulse of colloids is injected at the inlet of a microchannel. We assume that they all have the same initial lateral position $x_{i}$, but are spread along the axial direction according to a Gaussian distribution, as shown in the inset of Figure 10, right. At time t=0 the center of the Gaussian is at $z_{i}=0$. Now, we ask which final lateral position $x_{f}$ the colloids attain when reaching the axial target position $z_{t}$ under the action of the optimal control force $f_{\mathrm{ctl}}^{*}\left(t\right)$. For the latter, we use the optimal force protocol calculated for the central initial position at $z_{i}=0$. It is switched on at t=0 and switched off at the time $T^{*}$ when the particle moving on the original optimized trajectory with $z_{i}=0$ has reached the axial target position $z_{t}$. Because all particles start on the same initial lateral position $x_{i}$ at t=0, they move on replicas of the opimized trajectory, but shifted along the *z* direction by $z_{i}$. Colloids with $z_{i}>0$ precede the original optimized trajectory and therefore experience the control force until they have reached $z_{t}$. However, for colloids lagging behind the optimized trajectory ($z_{i}<0$), we simply turn off the control force once the optimized time period $T^{*}$ has passed and wait until they have reached $z=z_{t}$. During this time, the lateral motion is completely determined by inertial focusing without any Saffman force, where the focusing position is $x_{\mathrm{eq}}^{0}$. This allows for two scenarios: if the lateral target position $x_{t}$ is closer to the channel center than the focusing position ($x_{t}<x_{\mathrm{eq}}^{0}$), then we know from Section 4.2 that the optimal trajectory approaches the target from above. Therefore, the preceding and lagging colloids will both reach a final lateral position $x_{f}>x_{t}$. In contrast, for targets closer to the channel wall than the focusing position ($x_{t}>x_{\mathrm{eq}}^{0}$), both leading and lagging colloids end up closer to the center than the target ($x_{f}<x_{t}$).

The qualitative description is confirmed by Figure 10, left, where we plot the final lateral positions $x_{f}$ versus $z_{i}$ for different target positions $x_{t}$. They follow piecewise linear functions, where the two arms have different slopes, since different mechanisms determine the final positions of preceding and lagging colloids. Only for the target position $x_{t}=0.6$ do the final positions $x_{f}$ deviate from the linear course for very negative $z_{i}$. We note that the deviations of $x_{f}$ from the target remain small, even when the axial particle positions are spread over 30w to both sides of $z_{i}=0$.

Taking the Gaussian distribution of initial axial positions in the inset of Figure 10, right, with standard deviation $\sigma_{z}=10w$ and assuming the linear dependence $x_{f}\left(z_{i}\right)$ for the final lateral positions, one can readily write the distribution $p(x_{f})$ of final lateral positions. It is a superposition of two Gaussian functions, where only one half is used from each Gaussian (see below). The resulting distributions for the different target positions are presented in Figure 10, right. Although the axial width of the initial distribution is ca. 50w, the final positions only deviate a little from the target position. The distribution $p(x_{f})$ for $x_{t}=0.4w$ (green curve) is the sharpest, since $x_{t}=0.4w$ is closest to the zero-force focusing position $x_{\mathrm{eq}}^{0}$. The distributions become broader when $x_{t}$ is moved towards the wall or the channel center, respectively. Thus, here we demonstrate that a pulse of colloidal particles fairly spread in the axial direction can be focused into one target position at the channel outlet using one control-force protocol for all of the particles.

At the end, we shortly present the derivation of the distribution $p(x_{f})$ of final positions at the channel outlet. Because particles with a specific initial axial position $z_{i}$ move to a specific $x_{f}$, one can directly derive $p(x_{f})$ from the distribution $p_{z}\left(z_{i}\right)$ and obtain:

(9) $$p\left(x_{f}\right)=p_{z}\left(f_{+}^{-1}\left(x_{f}\right)\right)|{\left(f_{+}^{-1}\right)}^{\prime }\left(x_{f}\right)|+p_{z}\left(f_{-}^{-1}\left(x_{f}\right)\right)\left|{\left(f_{-}^{-1}\right)}^{\prime }\left(x_{f}\right)\right|.$$

Here, $x_{f}=f_{\pm }\left(z_{i}\right)=a_{\pm }z_{i}+x_{t}$ is the piecewise linear function from fitting the curves in Figure 10, left, $z_{i}=f_{\pm }^{-1}\left(x_{f}\right)$ is its inverse function, and ${\left(f_{\pm }^{-1}\right)}^{\prime }\left(x_{f}\right)=1/a_{\pm }$. Taking a Gaussian distribution for $p_{z}\left(z_{i}\right)$, the final distribution $p(x_{f})$ is a sum of two shifted and rescaled Gaussians with means at $x_{t}$. However, because the value range of $f_{\pm }$ is either $\left(-\infty ,x_{t}\right]$ or $\left[x_{t},\infty \right)$, the end result is a sum of two half-normal distributions, either to the left ($x_{t}>x_{\mathrm{eq}}$) or to the right ($x_{t}<x_{\mathrm{eq}}$) of the mean $x_{t}$ of the full Gaussian. This is readily seen in Figure 10, right. To have a quantitative measure for the width of the distribution $p(x_{f})$, we calculate its mean value:

(10) $$\mu =x_{t}\pm \frac{\sigma_{z}}{\sqrt{2\pi }}\left(\right|a_{+}\left|+\right|a_{-}\left|\right),$$

where the plus sign applies to $x_{t}<x_{\mathrm{eq}}^{0}$ and vice versa. The deviation from $x_{t}$ provides a measure for the width of $p(x_{f})$. It is determined by the slopes $a_{\pm }$ of the linear fits to $x_{f}=x_{f}\left(z_{i}\right)$. Because they are also small, the width is small and it decreases when $x_{t}$ approaches $x_{\mathrm{eq}}^{0}$. Ultimately, this small width comes from the fact that drift velocities in lateral channel direction are much smaller than the axial flow velocity. This means that inertial transport is much weaker than axial transport due to Poiseuille flow.

#### 4.3.2. Separation of Particles

**Figure 11:**
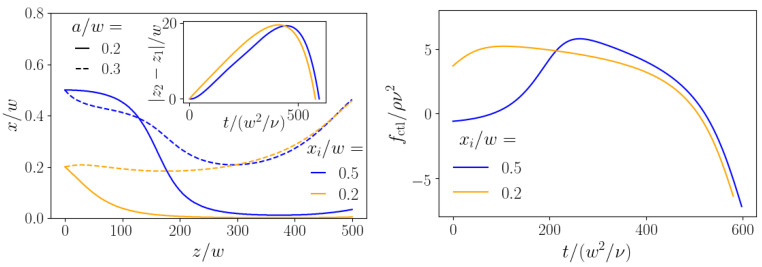
Maximizing the lateral distance of two particles with different radii a/w=0.2 and 0.3 using the same control-force protocol $f_{\mathrm{ctl}}^{*}\left(t\right)$. The particles both enter at the same inlet at $x_{i}$ and travel an axial distance with optimal value $z_{t}=500w$ during time $T^{*}$. (**Left**): trajectories of the two particles for both initial conditions. Inset: axial separation over time of the two particles. (**Right**): optimal control-force protocols for the two initial lateral positions $x_{i}=0.2w$ and 0.5w.

In the end, we examine the case where two particles of different size are steered using the same control-force protocol. Thus, we assume here that both particle types experience the same external force independent of their sizes. From Figure 3, we already know that this is possible: a properly chosen constant axial force can drive the smaller particle to the center while the larger particle stays at a finite distance from the center. Here, we aim to maximize the lateral distance after both particles have traveled the distance $z_{t}$ in axial direction. At the end of Section 3.3, we already formulated the appropriate cost functional for maximizing the lateral distance between both particles. In Figure 11, we show the resulting trajectories (left) and force protocols (right) for two particles with radii $a_{1}=0.2w$ and $a_{2}=0.3w$ and the axial target $z_{t}=500w$. Without control force, these particles would arrive at very similar positions, because their zero-force equilibrium positions are very close to each other. We present results for two cases where both particles start at the same initial position at either $x_{i}=0.2w$ or $x_{i}=0.5w$. Again, we assume that they do not interact. Interestingly, the control-force protocols for both cases look rather different in the beginning. However, in both cases, the smaller particle (solid lines in Figure 11, left) is pushed towards the centerline, while the larger particle (dashed lines) moves towards the channel wall during the second half of the trajectories. The separation reached at the end is Δx=0.45w for $x_{i}=0.2w$ and Δx=0.43w for $x_{i}=0.5w$, which is not attainable with any passive method.

**Figure 12:**
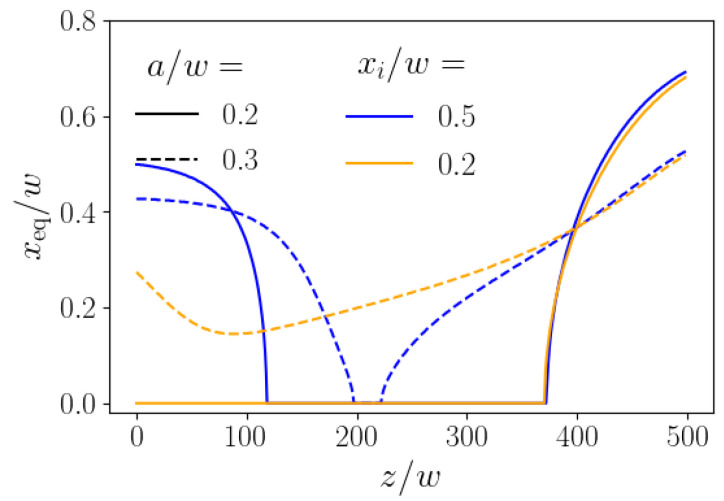
Instantaneous stable fixed points resulting from the applied forces in Figure 11 (left).

To develop a better understanding for the optimal control-force protocols of Figure 11, right, we show in Figure 12 the momentary stable fixed points for the smaller and larger particles corresponding to the momentary axial control force, when the particles are at position *z*. The path of the momentary fixed points reflects the particle trajectories of Figure 11, left, where the algorithm attempts to steer the smaller particle (solid lines) to the channel center and the larger particle (dashed lines) towards the wall. For initial position $x_{i}=0.5w$ (blue lines), the fixed point at zero control force is closer to the channel center (around 0.4w); therefore, the control force close to zero is sufficient to move, in particular, the smaller particle towards the center. Subsequently, it rises noticeably, bringing the smaller particles to the center, as documented by the course of the momentary fixed point. In contrast, the initial position $x_{i}=0.2w$ (orange color) is already closer to the channel center. Thus the control force is switched on immediately to push the smaller particle (orange solid line) to the channel center, where the momentary fixed point is located. As we know from Figure 3, left, the fixed point of the large particle does not react so strongly to the control force. It is only shifted towards the center, but hardly reaches it. Nevertheless, this initial behavior causes the minima in the dashed trajectories of Figure 11, left. Afterwards, in both cases the axial control force identified by our algorithm becomes strongly negative and the momentary fixed points are pushed towards the wall, even stronger for the smaller particles. Nevertheless, because the lift force scales with the particle radius $a^{2}$, the larger particles are pushed towards the wall, while the smaller particles stay close to the channel center and hardly move away from it (see Figure 11, left).

## 5. Conclusions

We applied concepts from optimal control theory to a setup from inertial microfluidics and managed to precisely steer single particles from a microchannel inlet to an outlet while using a time-dependent axial force, which controls the lateral inertial lift force via the Saffman effect. Our results show that the optimal control force exploits conventional inertial migration, since, in the beginning, it is zero, so that the particle drifts towards its lateral equilibrium position. Only then the control force is switched on, so that the particle is pushed towards a target position. Because of this property, steering with an optimized control force is cheaper than a strategy where a constant axial force is used for steering. Additionally, the optimal-control strategy can be implemented for different channel lengths, which makes this approach versatile. We also used the optimal control-force protocol for a single particle to demonstrate that even a pulse of particles spread along the channel axis can be steered to a target with only a small spread around the exact target position. Finally, we showed how a single optimized control-force protocol can separate particles of similar radii $a_{1}=0.2w$ and $a_{2}=0.3w$. The lateral distances reached for a channel length of 500w and different initial positions are considerably larger than a passive strategy could achieve.

It would be interesting to explore different channel geometries in the future, such as rectangular cross sections with different aspect ratio or triangular cross sections [37], because they strongly influence the locations of the fixed points of the inertial lift force. For triangular microchannels, this would require determining the lift-force profile in the cross-sectional plane and not just on one axis.

We consider particle steering by an optimal axial control force as an innovative method for targeting precise positions at the channel outlets, which will then have implications for particle separation and filtration. We hope that our work stimulates future efforts towards an experimental realization. As we outlined above, to realize the axial control force, we suggest the use of electric fields in combination with electrophoresis, which has already been applied in experiments on the Saffman effect and inertial migration [16,17] and also studied in theory [18]. Typically, micron-sized particles can exhibit electrophoresis and it is also realized for biological cells [38].
